# High-Strength Ultra-Fine-Grained Hypereutectic Al-Si-Fe-X (X = Cr, Mn) Alloys Prepared by Short-Term Mechanical Alloying and Spark Plasma Sintering

**DOI:** 10.3390/ma9120973

**Published:** 2016-11-30

**Authors:** Filip Průša, Markéta Bláhová, Dalibor Vojtěch, Vojtěch Kučera, Adriana Bernatiková, Tomáš František Kubatík, Alena Michalcová

**Affiliations:** 1 Department of Metals and Corrosion Engineering, University of Chemistry and Technology Prague, Technická 5, 166 28 Prague, Czech Republic; blahovar@vscht.cz (M.B.); vojtechd@vscht.cz (D.V.); kucerao@vscht.cz (V.K.); bernatia@vscht.cz (A.B.); michalca@vscht.cz (A.M.); 2 Institute of Plasma Physics, Czech Academy of Sciences, Za Slovankou 1782/3, 182 00 Prague, Czech Republic; kubatik@ipp.cas.cz

**Keywords:** mechanical alloying, spark plasma sintering, microstructure, mechanical properties

## Abstract

In this work, Al-20Si-10Fe-6Cr and Al-20Si-10Fe-6Mn (wt %) alloys were prepared by a combination of short-term mechanical alloying and spark plasma sintering. The microstructure was composed of homogeneously dispersed intermetallic particles forming composite-like structures. X-ray diffraction analysis and TEM + EDS analysis determined that the α-Al along with α-Al_15_(Fe,Cr)_3_Si_2_ or α-Al_15_(Fe,Mn)_3_Si_2_ phases were present, with dimensions below 130 nm. The highest hardness of 380 ± 7 HV5 was observed for the Al-20Si-10Fe-6Mn alloy, exceeding the hardness of the reference as-cast Al-12Si-1Cu-1 Mg-1Ni alloy (121 ± 2 HV5) by nearly a factor of three. Both of the prepared alloys showed exceptional thermal stability with the hardness remaining almost the same even after 100 h of annealing at 400 °C. Additionally, the compressive strengths of the Al-20Si-10Fe-6Cr and Al-20Si-10Fe-6Mn alloys reached 869 MPa and 887 MPa, respectively, and had virtually the same values of 870 MPa and 865 MPa, respectively, even after 100 h of annealing. More importantly, the alloys showed an increase in ductility at 400 °C, reaching several tens of percent. Thus, both of the investigated alloys showed better mechanical properties, including superior hardness, compressive strength and thermal stability, as compared to the reference Al-10Si-1Cu-1Mg-1Ni alloy, which softened remarkably, reducing its hardness by almost 50% to 63 ± 8 HV5.

## 1. Introduction

Increasing production of specialized products creates demand for advanced materials with improved mechanical properties as compared to the conventionally cast alloys used today. Therefore, routes to increase hardness, strength, thermal stability, corrosion resistance and other properties have attracted increasing attention. Rapid solidification (RS) [1,2,3,4,5] or severe plastic deformation (SPD) [6,7,8,9] are well-known processes but are still capable of producing alloys of known chemical compositions with brand new and outstanding properties. Among these processes, mechanical alloying (MA) is capable of producing alloys with a refined microstructure, including a nanocrystalline particle size, an extended solid solubility range, quasi-crystalline or amorphous phases, disordered intermetallic compounds and other features [10,11,12]. This process is well known for its easy reproducibility, allowing production of large amounts of alloy with desired properties while maintaining a low cost of production. From the first minutes of mechanical alloying, cold-welding of powder particles allows a limited elemental diffusion sufficient to create intermediate phases, followed by deformation hardening. Further increases in MA time lead to further hardening until it reaches a saturation level where the speed of dynamic recovery competes with the speed of deformation hardening. In some cases, the amount of cold welding can be reduced by process control agents (PCAs), which are usually organic compounds (e.g., ethanol, methanol, hexane, stearic acid, oxalic acid and many others), used in amounts ranging from 0.3 to 4 wt % [10]. The PCA agents usually act as surfactants to inhibit the agglomeration that would otherwise negatively affect the MA. On the atomic scale, the PCA compound affects the tip of the microcrack while weakening the interatomic bonding and facilitating crack growth while it prevents the closing of newly formed surfaces by adsorbing on the surfaces (Rebinder’s effect). However, the reactivity of certain PCAs with MA alloys should be considered to prevent undesirable contamination [10]. Aluminum, nickel and copper are typical elements that are capable of reacting with the PCA during the MA to form different kinds of metallo-organic compounds or may even react explosively (e.g., titanium and zirconium with chlorinated PCAs) [10]. However, in some cases, the reaction between the MA material and the PCA can be beneficial due to the formation of homogeneously dispersed particles such as Al_4_C_3_ or TiN. The intentional presence of such “impurities” can be achieved by reactive milling where the process is performed in the presence of reactive solids, liquids, or gases, causing formation of oxides, nitrides or carbides [10]. It should be noted that MA is typically carried out for several tens of hours [11,13,14,15,16] and, therefore, the MA used in the present work belongs to the short-term operations since it was done for only eight hours.

The Al-Si based alloys are known for their wide utilization in the automotive industry mainly due to their good weight-to-strength ratio, excellent wear resistance, low thermal expansion coefficients and interesting electrical properties [17,18,19,20]. They are used in a wide range of other applications, such as marine casting; engine fittings and pistons; whole engine blocks, including cylinders and heads; transmissions; pump parts; impellers; and many others. However, their thermal stability is limited to temperatures that do not exceed 200 °C. Fe is the most common impurity in Al alloys, and it forms thermally stable intermetallic phases while widening the temperature limits for applications of the aluminum alloys [17,19,21,22,23]. Moreover, Fe in the range of 0.8 wt %–1.1 wt % is necessary for pressure die casting to prevent the melt from soldering to the steel cast die [21]. However, the nature of the newly formed intermetallic phases often degrades the mechanical properties of the alloys. Microstructural refinement down to the sub-micrometer or even to the nanoscale size is crucial to obtaining outstanding mechanical properties (e.g., hardness and strength) of these materials. Thus, an appropriate amount of alloying elements with a low diffusion coefficient in solid aluminum improves the thermal stability. Transition metal elements such as Fe, Cr, Mn and others are widely used for this purpose [20,21,23]. However, the presence of Fe in Al-Si-based alloys results in the formation of the common needle-like *β*-Al_5_FeSi (τ_6_) intermetallic phases with dimensions reaching up to several hundreds of micrometers [24], and their presence strongly degrades the mechanical properties of such materials [17,24]. Due to high brittleness of *β*-Al_5_FeSi (τ_6_), it acts as a strong stress concentrator while its three-dimensional platelet or needle-like morphology causes the formation of voids due to the presence of interdendritic channels suppressing the melt flow [25,26]. More importantly, the dimensions of the harmful *β* phase increases with the content of Fe in the alloy [24]. Additional main intermetallic phases in the Al-Si-Fe system are θ-Al_13_Fe_4_, α-Al_8_Fe_2_Si (τ_5_), δ-Al_4_FeSi_2_ and γ-Al_3_FeSi, all with mostly needle-like morphology [26,27]. In total, there are twelve Fe-based stable phases and one metastable phase in the Al-Si-Fe system. It is evident that the presence of these large phases with continuous character is undesirable. On the other hand, the presence of the previously mentioned Mn diminishes the negative impact of the second phases by transforming the large and brittle phases into less harmful Chinese-script phases such as the α-Al_15_(Fe,Mn)_3_Si_2_ phase while reducing their overall dimensions [19,21,24,28,29,30]. Cai et al. [19] have found that the addition of identical amounts of both Cr and Mn differ in their efficiency to modify the harmful *β* phase, where Cr was much more effective than Mn. Additionally, the wear properties of Al-Si-Fe alloy containing the more beneficial α-Al_15_(Fe,Mn)_3_Si_2_ phase is highly improved [17,20]. The amount of Mn is specified in the ANSI/ASTM B108-78 and should be equal to one half of the total Fe amount, as is suggested in several works [17,21,30,31].

Solute strengthening and the formation of fine and homogeneously dispersed thermally stable phases are therefore beneficial. For these purposes, rapid solidification [32,33] methods and severe plastic deformation methods including accumulative roll bonding, high-pressure torsion or mechanical alloying [25,34] are widely used.

In the present work, Al-Si-Fe-Cr-Mn alloys were successfully prepared by short-term MA and compacted by spark plasma sintering (SPS) to maximize retention of the beneficial character of the microstructure. It was shown that the alloys reached ultra-high hardness and that their compressive strength was superior to the cast Al-12Si-1Cu-1Mg-1Ni alloy used as the reference material. Additionally, it was shown that hypereutectic Al-Si-based alloys containing up to 10 wt % of transition elements can be processed while obtaining quite unique properties, including excellent thermal stability.

## 2. Results

### 2.1. Microstructure and Phase Compositions

**Figure 1:**
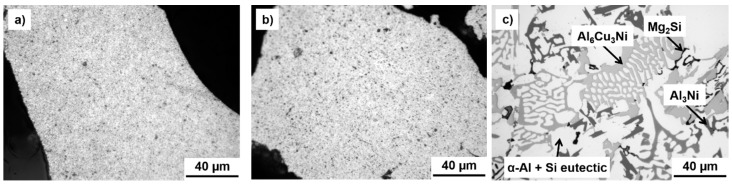
LM (light microscope) of the short-term MA (mechanical alloying): (**a**) Al-20Si-10Fe-6Cr; (**b**) Al-20Si-10Fe-6Mn powder alloys and (**c**) of the as-cast reference Al-12Si-1Cu-1Mg-1Ni alloy.

The LM micrographs of the prepared MA powder samples are shown in Figure 1a,b. It is clearly visible that the samples were composed of fine intermetallic phases homogeneously dispersed in the matrix, forming an almost composite-like material. The fine nature of the phases present resulted in good mechanical properties and good thermal stability, as will be shown further in the text. On the other hand, the microstructure of the as-cast reference Al-12Si-1Cu-1Mg-1Ni alloy (Figure 1c) contained sizable phases with dimensions reaching several tens of micrometers. The compositions of the phases present in this alloy were identified by SEM + EDS point analysis as α-Al, Al_3_Ni, Al_6_Cu_3_Ni and Mg_2_Si as shown in our previous work [25].

**Figure 2:**
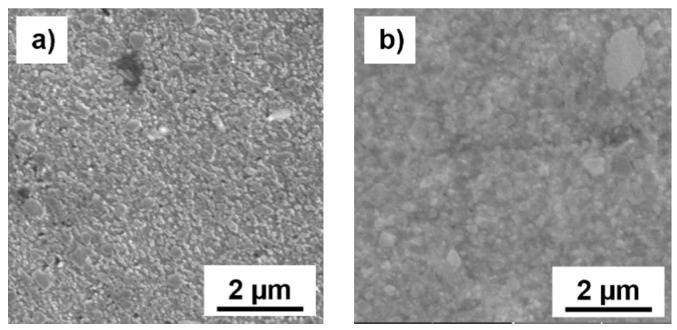

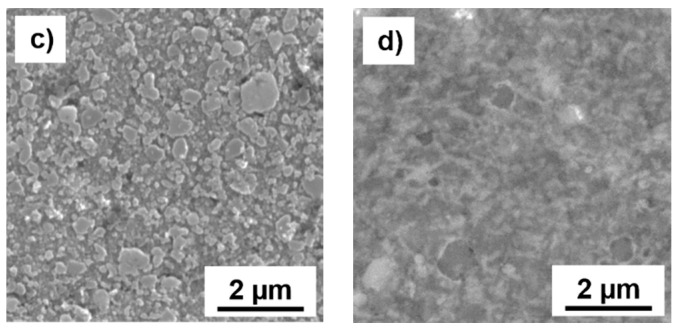
Detailed SEM micrographs of: (**a**,**b**) Al-20Si-10Fe-6Cr; (**c**,**d**) Al-20Si-10Fe-6Mn alloys prepared by short-term MA (**a**,**c**) and compacted by SPS (**b**,**d**).

More detailed SEM micrographs (Figure 2a,c) reveal the fine nature of the intermetallic phases that were almost circular in aspect with sub-micrometer dimensions. This beneficial microstructure remained even after compaction via SPS performed at the relatively high temperature of 500 °C (Figure 2b,d).

**Figure 3:**
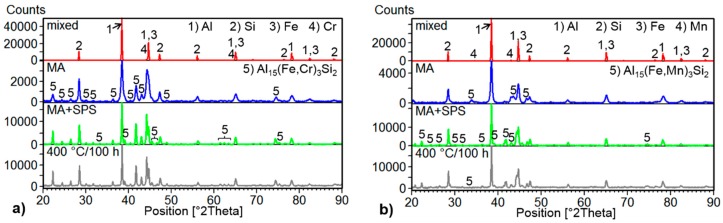
XRD patterns of the: (**a**) Al-20Si-10Fe-6Cr; (**b**) Al-20Si-10Fe-6Mn alloys at different stages of preparation including as mixed pure powders, MA powders, MA + SPS compact samples and MA + SPS compact samples annealed at 400 °C for 100 h.

The X-ray diffraction analysis shown in Figure 3 confirmed the presence of α-Al in both MA + SPS compacted alloys, supporting the prediction of supersaturated solid solution formation as well as the presence of intermetallic phases and primary Si. The intermetallic phases identified in the XRD analysis of the Al-20Si-10Fe-6Cr and Al-20Si-10Fe-6Mn alloys were Al_15_(Fe,Cr)_3_Si_2_ and Al_15_(Fe,Mn)_3_Si_2_, respectively. Moreover, the results displayed in Figure 3 confirmed the formation of intermetallic phases already after 8 h of MA as well as grain refinement and formation of a supersaturated solid solution. The grain refinement and formation of a supersaturated solid solution is clearly visible by a more detailed look at the FWHM (Full Width at Half Maximum) values and by the peak shift of the Al (111) to higher diffraction angles (see Table 1). For the Al-20Si-10Fe-6Cr alloy, the Al (111) peak changed from an initial 2θ angle and an FWHM value of 38.379° and 0.140, respectively, to 38.427° and 0.264 following MA. The 2θ angle and FWHM value for the Al-20Si-10Fe-6Mn alloy changed from 38.408° and 0.131, respectively, before MA, to 38.449 and 0.286 following MA. Such changes of the diffraction angles and of the FWHM values are attributed to the formation of supersaturated solid solutions and to significant grain refinement, respectively. However, it should be noted that the change of the FWHM value is also partially caused by the lattice distortion induced during MA. The same behavior was also observed in the X-ray peaks of Si, suggesting its refinement during MA.

**Figure 4:**
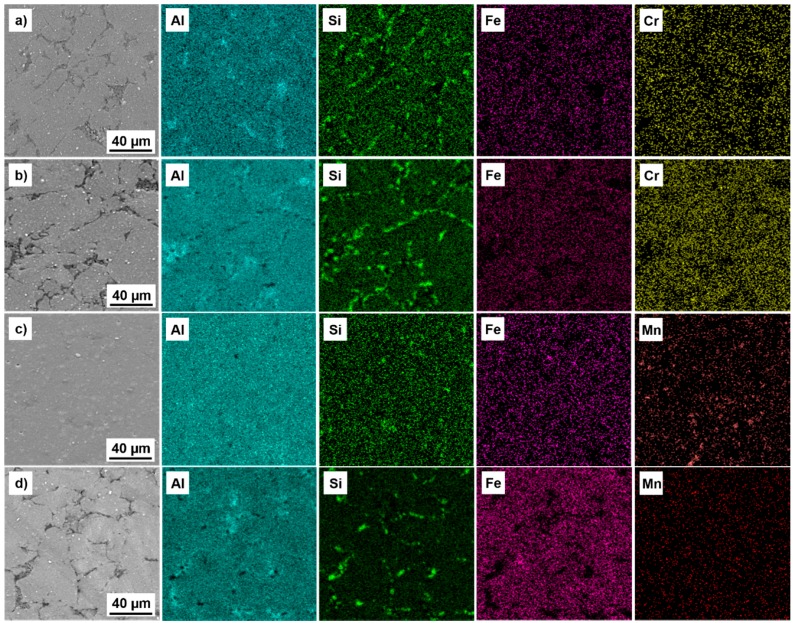
SEM + EDS maps of the: (**a**,**b**) Al-20Si-10Fe-6Cr; and (**c**,**d**) Al-20Si-10Fe-6Mn alloys. (**a**,**c**) Compact samples prepared by the combination of MA + SPS; and (**b**,**d**) the same materials after 100 h annealing at 400 °C.

During compaction via SPS, a slight coarsening of the Si particles was observed and this is clearly demonstrated by the tendency to narrow the characteristic diffraction patterns of Si (see Figure 3). The same behavior is clearly demonstrated by the change in the FWHM values of the Al (111) peaks, corresponding to relaxation of lattice stress and, more importantly, to grain coarsening. Compared to Al, the intermetallic phases were thermally stable and retained their initial dimensions (Figure 2b,d). Moreover, the same behavior was observed after annealing at 400 °C for 100 h (Figure 4). The intermetallic phases were thermally stable even when exposed to temperatures far beyond the common use conditions for aluminum alloys (Figure 3 and Figure 4).

**Figure 5:**
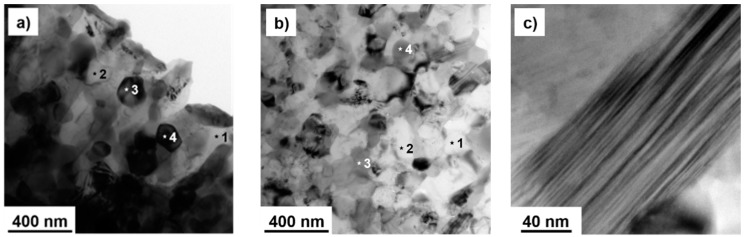
Bright field TEM micrographs of MA + SPS: (**a**) Al-20Si-10Fe-6Cr; and (**b**) Al-20Si-10Fe-6Mn alloys with marked points of EDS X-ray analysis (TEM + EDS); and (**c**) more detailed view of the stacking fault present in intermetallic phases of the Al-20Si-10Fe-6Mn alloy.

The phase compositions mentioned previously was also confirmed by the more detailed TEM + EDS analysis (Figure 5, Table 2). Both of the MA + SPS alloys were composed of α-Al (light) and intermetallic (dark) phases. For the Al-201Si-10Fe-6Cr alloy, the dimensions of the α-Al and the intermetallic phases were 121 ± 25 nm and 120 ± 25 nm, respectively. For the Al-20Si-10Fe-6Mn alloy, those phases were 128 ± 38 nm and 115 ± 29 nm, respectively. The α-Al grains were light with almost circular geometry, suggesting a low concentration of lattice defects due to partial recovery during the compaction via SPS. In comparison, the intermetallic phases were characterized by the presence of higher amount of lattice defects, including stacking faults, that manifested themselves as parallel lines in the grains interior (Figure 5c). The presence of stacking faults was confirmed also in the MA + SPS Al-20Si-16 and Al-10Si-21Fe alloys investigated in our previous work [25].

The results of point analysis of chemical compositions marked in Figure 5a,b are shown in Table 2. Both MA + SPS alloys were composed of supersaturated solid solutions that contained up to 2 at % of transition elements. The intermetallic phases were identified based on the present results as Al_15_(Fe,Cr)_3_Si_2_ and Al_15_(Fe,Mn)_3_Si_2_ and further confirmed by the X-ray diffraction analyses, which are shown in Figure 3.

### 2.2. Mechanical Properties

**Figure 6:**
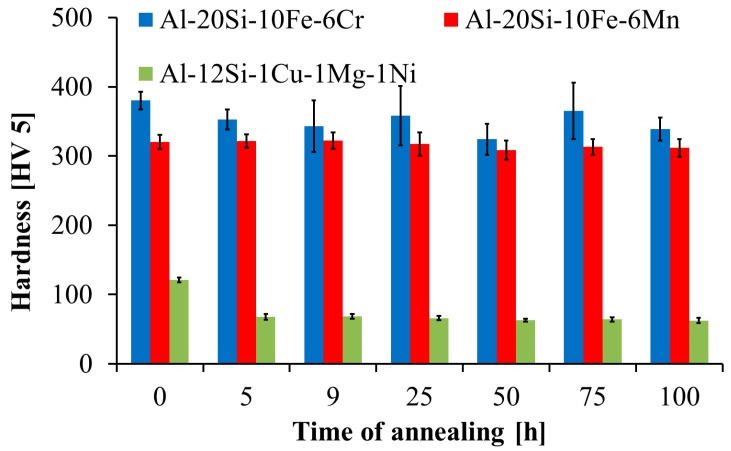
Thermal stability of tested MA + SPS alloys expressed as hardness (HV5) as a function of annealing time at 400 °C.

The hardness of the prepared MA + SPS alloys was evaluated, and the thermal stability of the alloys was evaluated by measuring the hardness as a function of annealing time at 400 °C. The results are shown in Figure 6 and summarized in Table 3. The highest initial hardness after compaction was achieved by the Al-20Si-10Fe-6Cr alloy, which reached 380 ± 7 HV5, followed by the Al-20Si-10Fe-6Mn alloy, with a hardness of 320 ± 6 HV5. On the other hand, the hardness of the reference as-cast alloy was more than three times lower (121 ± 11 HV5) compared to the best result of the Al-20Si-10Fe-6Cr alloy. During annealing at 400 °C, the hardness of the prepared MA + SPS alloys remained at their high initial hardness, confirming their good thermal stability. In comparison, the reference as-cast Al-12Si-1Cu-1Mg-1Ni alloy drastically softened, with its hardness reduced by almost 50% to 63 ± 8 HV5 following annealing.

**Figure 7:**
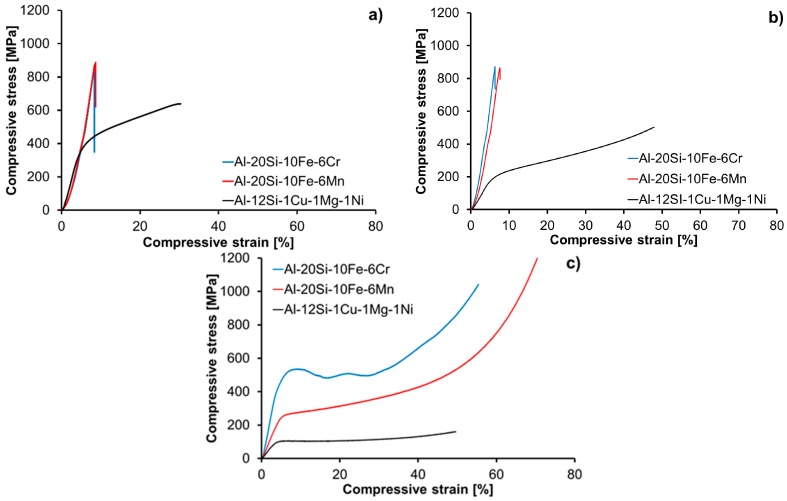
Compressive stress–strain curves of the MA + SPS alloys measured under different conditions: (**a**) laboratory temperature; (**b**) after 100 h of annealing at 400 °C; and (**c**) at 400 °C (10 min of tempering).

The compressive stress–strain curves of MA + SPS samples measured at laboratory temperature, at laboratory temperature after 100 h of annealing at 400 °C and at the elevated temperature of 400 °C (10 min of tempering) are shown in the Figure 7 and summarized in Table 3. As shown in these plots, the MA + SPS alloys outperformed the reference Al-12Si-1Cu-1Mg-1Ni alloy. Regarding the laboratory temperature stress–strain tests, the as-compacted and annealed samples (Figure 7a,b) showed an almost identical compressive strengths (CS) of 880 MPa, exceeding the CS of the reference Al-12Si-1Cu-1Mg-1Ni alloy. The compressive test at 400 °C again showed the superiority of the prepared MA + SPS alloys compared to the reference material. One can see that the MA + SPS Al-20Si-10Fe-6Cr alloy exhibited the highest CYS of 409 MPa, a value that was four times higher than the CYS of the reference material. The MA + SPS Al-20Si-10Fe-6Mn alloy exhibited a lower, but still highly acceptable, CYS of 232 MPa. During the test at elevated temperature, both of the MA + SPS alloys showed an increased ductility that reached more than 50%.

## 3. Discussion

### 3.1. Microstructure

Compared to the results published in our previous work [25], the addition of Cr or Mn resulted in a finer microstructure where the grain size of the phases reached dimensions of approximately 130 nm. It was probably caused by the ability of Cr [4,19,35] and Mn [19,30,36,37,38] to modify the morphology of the commonly observed *β*-Al_5_FeSi_2_ phases into the more beneficial α-Al_15_(Fe,Mn,Cr)_3_Si_2_ intermetallic phases while the content of Si remained the same as it was in the Al-20Si-16Fe alloy [25]. The refined microstructure positively influenced the mechanical properties, specifically the hardness, the CS, or the CYS. Therefore, the addition of Cr or Mn showed itself as a new, effective method to further refine the microstructure. Regarding the modification ability during slow solidification techniques, the amount of Mn should be at least one half of the total weight amount of Fe [19,30,31], although some scientific publications reported 3 wt % of Mn as efficient [36,37]. Therefore, the addition of 6 wt % of Cr or Mn (representing 60% of the amount of Fe) was high enough to fulfill this condition. The phase composition given by the XRD analysis (Figure 3) and by the TEM + EDS results (Table 2) confirmed the presence of the *β*-Al_15_(Fe,Cr)_3_Si_2_ and *β*-Al_15_(Fe,Mn)_3_Si_2_ phases that were previously observed by others [17,19,20,21,22,30]. In these intermetallic phases, the atoms of Fe, Mn and Cr are able to change their positions in the crystallographic lattice due to their very similar atomic radii of 0.128 nm, 0.137 nm and 0.136 nm [39], respectively. Since these phases belong to hard phases, their fine nature with dimensions below 130 nm beneficially affected the mechanical properties including the Vickers hardness and/or CS and CYS. More importantly, the fine nature of the intermetallic α phases and their morphology can further increase the CS at elevated temperatures [30] and improve the wear behavior of these materials [20]. The reason for this can be found in the much rougher interface of the α phases with the α-Al matrix and by their better bonding that decreases the possibility of crack formation at the interface and subsequent crack growth [20,40]. The TEM + EDS results (Table 2) showed the presence of an α-Al solid solution than contained up to 2 at % of alloying elements, due to the similar atomic radii of Al (0.143 nm), Fe, Cr and Mn that effectively enhanced the dissolution.

Moustafa reported the negative effect of increasing the Fe content (in the range of 0.2 wt %–2.5 wt %) on increasing the growth of the harmful *β*-Al_5_FeSi phase in his work on the cast Al-11Si-xFe (wt %) alloys [24]. The dimensions of the *β* phase increased from 50 ± 10 µm to 720 ± 75 µm in length and from 1 ± 0.2 µm to 5.7 ± 0.6 µm in width. Moreover, he focused on modification of the eutectic Si particles by adding Sr, which surprisingly resulted in a further increase of the dimensions of this harmful phase up to 950 ± 45 µm in length and 6.1 ± 0.5 µm in width. From this point of view, the MA + SPS alloys investigated in this work showed a well-refined microstructure that contained both the α-Al and the Fe intermetallic phase with dimensions below 130 nm. This was caused by the MA process that is capable of reducing the dimensions of the observed phases down to the sub-micrometer or even to the nanoscale sizes, although the addition of Mn and Cr could contribute to the grain refinement as well.

During the annealing at 400 °C (see Figure 4), the microstructure coarsened, in particular the Si primary particles. In comparison, the intermetallic phases remained in their original shape and dimensions, supporting the prediction of increased high temperature stability. More importantly, these intermetallic phases are thermally stable even when a T6 heat treatment, including solution annealing at an extreme temperature of 510 °C for 7 h, was used during the manufacturing processes [22]. A similar observation was found in our investigations where the intermetallic phases remained in their initial dimensions even after 100 h of annealing at 400 °C. One can see from Figure 4 that the main microstructural change was the coarsening of the primary Si particles that could be explained by the much higher diffusion coefficient of Si in solid Al as compared to that of Fe, Cr and Mn. Compared to that, the microstructure of the reference as-cast Al-12Si-1Cu-1Mg-1Ni alloy coarsened significantly, mostly due to the higher values of the diffusion coefficients of Cu, Mg and Ni, as compared to the Fe, Cr and Mn used in the investigated MA + SPS alloys [25].

### 3.2. Mechanical Properties

To date, there has been a lack of reports describing the mechanical properties of hypereutectic Al-Si based alloys with the high Fe, Cr and Mn contents used in this study. The majority of authors published results from tensile tests to describe the mechanical properties. Nevertheless, the compressive behavior has informational value as well. Only compressive stress–strain tests were performed on the MA + SPS compact samples.

Both of the MA + SPS alloys showed a higher CS compared to the reference as-cast Al-12Si-1Cu-1Mg-1Ni alloy (Table 3, Figure 7a). The highest CS of 887 MPa was observed for the Al-20Si-10Fe-6Mn alloy, closely followed by CS of 869 MPa for the Al-20Si-10Fe-6Cr alloy. Such results are significantly higher than the strengths of high-strength wrought aluminum alloys strengthened by precipitation hardening, which typically reach CS values in the range of 550 MPa–600 MPa [11]. On the other hand, the reference as-cast alloy showed a CS of only 680 MPa. The lack of plasticity of both the MA + SPS alloys tested at laboratory temperature was caused by the fine nature of the α-Al and of the α-Al_15_(Fe,Cr)_3_Si_2_ or Al_15_(Fe,Mn)_3_Si_2_ grains. However, the plasticity could be theoretically increased by modifying the SPS process, particularly by intentionally coarsening the α-Al grains by using a higher heating rates during SPS [41] and/or by increasing the volume fraction of low-angle grain boundaries (LABs) achieved, for example, mostly by ECAP or hot extrusion after SPS [42].

More importantly, the MA + SPS alloys showed excellent thermal stability during annealing at 400 °C, which resulted in almost zero change of CS (Table 3, Figure 7b) following annealing. In comparison, the reference as-cast alloy softened remarkably during annealing, reducing the CS from 680 MPa to 498 MPa (Table 3, Figure 7b). The compressive stress–strain test at 400 °C confirmed again the superior strength of the prepared MA + SPS alloys compared to the as-cast reference material (see Table 3, Figure 7c). This behavior of the MA + SPS alloys was produced by their fine microstructure, confirming the Hall-Petch relationship; solid solution strengthening (see Table 2); and by the high volume fraction of hard and thermally stable intermetallic phases that can effectively hinder grain boundary sliding and dislocation gliding. Additionally, the MA + SPS alloys showed an enormous increase in ductility, reaching several tens of percent at 400 °C (Figure 7c), which is very important for potential applications of the MA + SPS alloys at elevated temperatures.

The MA + SPS Al-20Si-10Fe-6Cr alloy showed the highest hardness at laboratory temperature of 380 ± 7 HV5 followed by the second best result of 320 ± 6 HV5 for the Al-20Si-10Fe-6Mn alloy. Such a high hardness was caused by a high volume fraction of hard intermetallic phases whose dimensions did not exceed 130 nm. Further, the MA + SPS alloys showed excellent thermal stability, with almost zero change in hardness during the annealing at 400 °C for 100 h. Recently, Bidmeskhi et al. [17] reported in his work focused on as-cast Al-17.5Si-xFe alloys (x = 0.4 wt %–1.8 wt %) an increase of hardness from 115 ± 0.91 HB to 130 ± 0.23 HB by changing the concentration of Fe (from 1.2 wt % to 1.8 wt %) and by the addition of Mn (from 0 wt % to 0.8 wt %), which was added to modify the morphology of the *β*-Al_5_FeSi phase into a Chinese-script such as α-Al_15_(Fe,Mn)_3_Si_2_. This increase in hardness was further responsible for the better wear resistance, the best of which was found for the Al-17.6Si-1.8Fe-0.8Mn alloy. However, it should be noted that Bidmeskhi et al. prepared their alloys using conventional casting technology, allowing modification of the morphology of the harmful phases into the more beneficial Chinese-script morphology. Compared to that, our investigated alloys were prepared by high-energy MA, a process which combines cold welding with subsequent grain refinement, processes that are completely different from the results given in [17]. Lin et al. [22] focused theirs research on hypereutectic Al-17Si-2Fe-2Cu-1Ni alloy for which they used the combination of rheo-casting and assisted ultrasonic vibration process. The combination of these processing technologies resulted in a hardness of 141 HB, mainly caused by the presence of different intermetallic phases including the α-Al_15_(Fe,Mn)_3_Si_2_ and δ-Al_4_(Fe,Mn)Si_2_ phases with dimensions below 20 µm. The MA + SPS alloys investigated in the present work showed superior hardness (up to 380 HV5) compared to the results given by the other scientists.

The reference as-cast Al-12Si-1Cu-1Mg-1Ni alloy showed much lower mechanical properties compared to the MA + SPS alloys, especially the hardness, which reached a value of only one third of the MA + SPS alloys hardness. More importantly, the reference alloy remarkably softened during the annealing, reducing its initial hardness and CS of 121 ± 11 HV5 and 680 MPa, respectively, to 63 ± 8 HV5 and 498 MPa following annealing. This behavior can be explained by the higher diffusion coefficients of Mg, Ni and Cu in solid Al which allowed microstructure coarsening in the reference alloy as compared to the MA + SPS alloys.

## 4. Materials and Methods

**Figure 8:**
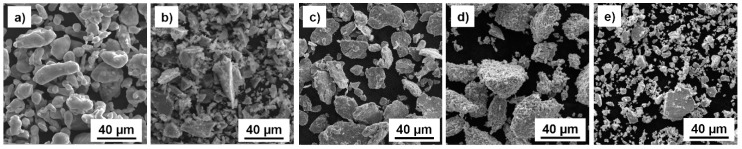
Morphology of: (**a**) Al; (**b**) Si; (**c**) Fe; (**d**) Cr; and (**e**) Mn powders used for short-term MA.

The investigated Al-Si-Fe-Cr-Mn alloys (see Table 4) were prepared by short-term MA and compacted via SPS. Pure powders of each element (purity of 99.9% or higher) were mixed together in appropriate amounts and placed into an AISI 420 steel mold together with milling balls. The mold was than sealed and flushed by argon to minimize oxidation during the MA. The morphology of the powders (Figure 8) varied, including almost cylindrical (Al powder), flake-like (Fe powder), and sharp-edged (Si and Mn powders). The average diameters of the powders used, with respect to the order of their appearance in Figure 8, was 10, 6, 7, 42 and 7 μm (Figure 8). It should be noted that the higher diameter of the Cr powder was due to the strong agglomeration of small particles that is clearly visible in Figure 8d.

**Figure 9:**
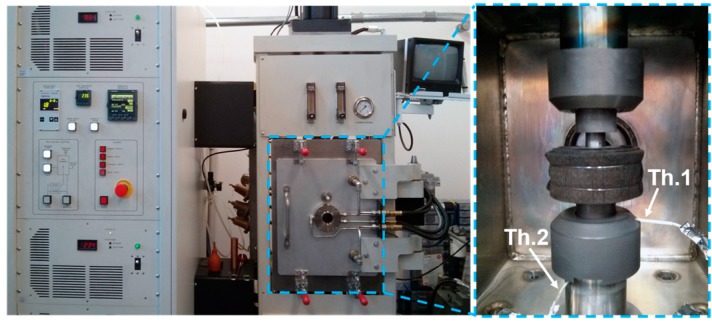
The SPS device Thermal Technology 10-4 with marked positions of thermocouples (Th.) for precise temperature control.

The short-term MA was performed in the Retsch PM100 alloying machine (Retsch PM 100, Haan, Germany) capable of performing high-energy mechanical alloying. The parameters of the process combined a rotation speed of 300 rpm, a ball-to-powder weight ratio of 30:1, and a change of the rotation direction every 30 min during the 8 h of the process. Prepared powders were consequently precompacted using a pressure of 230 MPa to obtain cylindrical billets measuring 19 mm in diameter and 5 mm in height. These semi-compact samples were placed into a graphite mold with an opening to accommodate thermocouples for precise temperature control (see Figure 9) during the compaction via SPS (model 10-4, Thermal Technology LLC, Santa Rosa, CA, USA).

**Figure 10:**
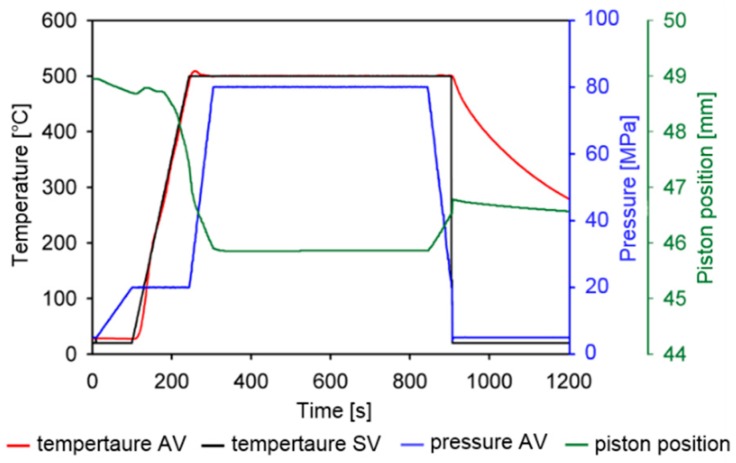
Scheme of the compaction process via SPS for the Al-20Si-10Fe-6Cr alloy (AV, actual value; SV, set point value).

At the start of the compaction process, the samples were pre-loaded with a pressure of 20 MPa to provide a good connection between the pressing pistons and the sample itself. The samples were then simultaneously heated at a rate of 200 °C∙min^−1^ to a final temperature of 500 °C, and consequently compressed to a total pressure of 80 MPa (see Figure 10). The sample remained at 500 °C for only 10 min to suppress undesirable microstructure coarsening. Height reduction of the samples by approximately 3 mm was observed (Figure 10) during SPS, indicating excellent particle-to-particle contact and elimination of free spaces between the powder particles.

The prepared compact samples were cut by a diamond blade mounted on a cutting machine Leco Vari/Cut VC-50 into the specimens used for further microstructural and mechanical investigations. For measurement of the mechanical properties, the samples were cut into cubes with a side length of 3.5 mm.

The samples for microstructural investigations were prepared by subsequent grinding on P180-P4000 abrasive SiC papers and polished using STRUERS OP-S suspension. Samples were etched in Keller’s reagent (2.5 mL HNO_3_, 1.5 mL HCl, 1 mL HF and 95 mL H_2_O). The microstructure was observed by light microscopy (LM, Olympus PME-3), scanning electron microscopy (SEM, Tescan Vega 3-LMU, Brno, Czech Republic, 20 kV, SE + BSE detectors) coupled with an energy dispersive X-ray spectrometer (EDS, INCA 350, Oxford Instruments, Abingdon, UK) and transmission electron microscopy (TEM, JEM 3010, JEOL, Croissy-sur-Seine, France, 200 kV, STEM mode) coupled with an EDS (Princeton Gamma-Tech Instruments Inc., Princeton, NJ, USA). To ensure good conductivity for the SEM + EDS analysis, the surface of samples was coated with a layer of gold (QUORUM Q150R ES) to a thickness of 2 nm. The phases and chemical compositions of the prepared samples were investigated by X-ray diffraction analysis (XRD, X’Pert Pro, PANalaytical, Almelo, The Netherlands, Cu Kα_1_ λ = 1.54059 Å) and by X-ray fluorescence spectroscopy (XRF, ARL 9400 XP, Thermo Fisher Scientific, Waltham, MA, USA), respectively. Based on the chemical compositions measured by the XRF analysis (see Table 4), the alloys will be referenced throughout the entire manuscript as Al-20Si-10Fe-6Cr and Al-20Si-10Fe-6Mn.

Prepared compact samples were then annealed at 400 °C in air to determine their thermal stability as measured by the change in Vickers hardness HV5 (load of 5 kg) during the 100 h of annealing. Moreover, the samples were compressive stress–strain tested at 400 °C after 10 min of tempering to ensure a homogeneous temperature distribution throughout the samples. The same tests were performed on the as-prepared compact samples and the samples that were annealed for 100 h at 400 °C. Compressive tests were performed with a strain speed of 1 mm∙min^−1^ on a LabTest 5.250SP1-VM machine (LaborTech, Ostrava, Czech Republic) equipped with a furnace allowing testing at temperatures up to 1000 °C.

The commercial, thermally stable Al-12Si-1Cu-1Mg-1Ni alloy, generally used for different engine parts manufacturing, was used as the reference material to compare the mechanical properties. It was provided by an external supplier and thermally treated using the T6 regime consisting of solution annealing (510 °C/5 h), water quenching and artificial ageing (230 °C/6 h) [25].

## 5. Conclusions

The present work demonstrates the positive influence of short-term 8 h MA on the resulting mechanical properties, if an appropriate compaction method is used. For this purpose, SPS was used as the compaction technique. The compact samples of both MA + SPS Al-20Si-10Fe-6Cr and Al-20Si-10Fe-6Mn alloys were characterized with a composite-like microstructure composed of supersaturated α-Al grains and of highly thermally stable α-Al_15_(Fe,Cr)_3_Si_2_ or α-Al_15_(Fe,Mn)_3_Si_2_ phases with dimension below 130 nm. Such a favorable microstructure resulted in excellent mechanical properties as well as enhanced thermal stability. Both of the investigated alloys showed ultra-high hardness, reaching values of up to 380 HV5 and a CS of almost 880 MPa, which remained almost unchanged even after annealing at 400 °C for 100 h. More importantly, the MA + SPS alloys significantly outperformed the reference as-cast Al-12Si-1Cu-1Mg-1Ni alloy that remarkably softened during the annealing at 400 °C, reducing its initial hardness by more than 50%. Both of the MA + SPS alloys showed significant increases in ductility during the compressive tests at 400 °C, which is highly beneficial for further processing.

## Figures and Tables

**Table 1:** Summary of the results of XRD analysis of the Al (111) peak in the investigated alloys at different stages of preparation (2θ, diffraction angle; FWHM, Full Width at Half Maximum).

Alloy (wt %)	Stage of Preparation	2θ (°)	FWHM
		Al (111)	Al (111)
Al-20Si-10Fe-6Cr	as mixed	38.379	0.140
	MA	38.427	0.264
	MA + SPS	38.433	0.195
	400 °C/100 h	38.458	0.194
Al-20Si-10Fe-6Mn	as mixed	38.408	0.131
	MA	38.449	0.286
	MA + SPS	38.445	0.195
	400 °C/100 h	38.458	0.195

**Table 2:** Chemical compositions (at %) given by the TEM + EDS point analysis of the present phases in MA + SPS Al-20Si-10Fe-6Cr and Al-20Si-10Fe-6Mn alloys.

Alloy (Preparation)	Element (at %)					Corresponding Phase
Point No.	Al	Si	Fe	Cr	Mn	
Al-20Si-10Fe-6Cr (MA + SPS)						
1	96.5	0.8	1.8	0.9	-	α-Al
2	97.2	0.9	1.2	0.7	-	α-Al
3	76.2	10.0	9.0	4.8	-	Al_15_(Fe,Cr)_3_Si_2_
4	77.5	9.1	8.7	4.7	-	Al_15_(Fe,Cr)_3_Si_2_
Al-20Si-10Fe-6Mn (MA + SPS)						
1	96.5	1.4	1.3	-	0.8	α-Al
2	96.0	0.9	1.7	-	1.4	α-Al
3	72.7	11.8	12.2	-	3.3	Al_15_(Fe,Mn)_3_Si_2_
4	76.1	9.4	10.4	-	4.1	Al_15_(Fe,Mn)_3_Si_2_

**Table 3:** Mechanical properties of the investigated MA + SPS alloys at laboratory temperature ^(LT)^ and at 400 °C (HV5, Vickers hardness; CS, compressive strength in MPa; CYS, compressive yield strength in MPa). (^a^ The CYS could not be determined for the MA + SPS alloys; ^b^ The HV5 and CS could not be determined during test at 400 °C).

Alloy	As-Prepared (LT)			Annealed at 400 °C/100 h (LT)			At 400 °C		
	HV5	CS	CYS ^a^	HV5	CS	CYS ^a^	HV5 ^b^	CSb	CYS
Al-20Si-10Fe-6Cr	380 ± 7	869	-	350 ± 10	870	-	-	-	409
Al-20Si-10Fe-6Mn	320 ± 6	887	-	312 ± 7	865	-	-	-	232
Al-12Si-1Cu-1Mg-1Ni	121 ± 11	680	430	63 ± 8	498	180	-	-	100

**Table 4:** Chemical compositions (wt %) of the MA + SPS alloys and of the reference as-cast AlSiCuMgNi material measured by XRF analysis.

Material (Preparation)	Element (wt %)									
	Ca	Cr	Cu	Fe	Mg	Mn	Ni	Si	Ti	Al
Al-20Si-10Fe-6Cr (MA + SPS)	-	5.8	-	10.4	-	-	-	20.3	-	Bal.
Al-20Si-10Fe-6Mn (MA + SPS)	-	0.3	-	10.1	-	6.1	-	20.2	-	Bal.
Al-12Si-1Cu-1Mg-1Ni (cast, T6 heat-treated)	0.1	0.1	1.2	0.2	1.0	0.2	0.9	11.8	0.1	Bal.
